# SIRT1: Protean roles at the nexus of health, disease, and therapeutics

**DOI:** 10.1002/ccs3.70086

**Published:** 2026-06-02

**Authors:** Brahim Chaqour

**Affiliations:** ^1^ Department of Molecular Ophthalmology University of Pennsylvania Perelman School of Medicine Philadelphia Pennsylvania USA

**Keywords:** aging, cardiovascular, mitochondria, neurodegeneration, signaling pathways, SIRT1, therapy

## Abstract

Silent mating type information regulators (sirtuins and SIRTs) have emerged as central nodes linking cellular metabolism to stress adaptation and diseases. SIRT1 stands out as a ubiquitously expressed, NAD^+^‐dependent deacetylase with remarkably broad and sometimes paradoxical functions. Beyond its canonical roles in chromatin regulation and DNA repair, SIRT1 regulates key pathways controlling metabolism, inflammation, circadian rhythms, mitochondrial biogenesis, mitophagy, immune response, and cellular survival. Growing evidence, including several studies published in JCCS, positions SIRT1 at the intersection of cardiovascular, metabolic, neurovascular, and oncogenic processes, highlighting both its biological complexity and therapeutic potential. Mechanistically, SIRT1 modulates through deacetylation, a diverse network of substrates including histones H3/H4, tubulin, BMAL1, p53, NF‐κB, FOXO, PGC‐1α, and eNOS, yet, its functional outcomes are highly context dependent. Although SIRT1 activation is often protective in metabolic and cardiovascular and neurovascular settings, its role in cancer remains dualistic, acting as both tumor suppressor and promoter. Similarly, its contribution to longevity remains unresolved, with conflicting results across experimental models. Pharmacological targeting of SIRT1, through compounds such as resveratrol and synthetic modulators, has generated considerable enthusiasm, but challenges related to specificity, bioavailability, and off‐target effects persist. Here, I discuss SIRT1 not simply as a therapeutic target but as a molecular “shapeshifter,” whose context‐dependent actions demand more precise biomarker‐guided strategies for clinical translation.

## Graphical abstract

SIRT1 serves as a central regulator linking metabolism, stress responses, and disease. It influences diverse processes, including transcriptional and posttranscriptional gene expression and protein activity, inflammation, mitochondrial function, and cell survival, through the deacetylation of multiple protein targets. While SIRT1 often plays protective roles in metabolic, cardiovascular, and neurovascular contexts, its effects are highly context dependent. SIRT1 activation holds promise for context‐driven therapeutic strategies.

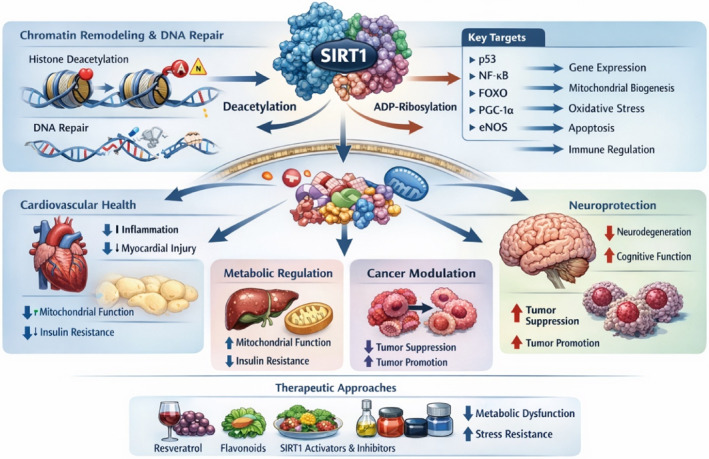

## 1 INTRODUCTION

Silent mating type information regulators (sirtuins) proteins (aka SIRTs) form a family of NAD^+^‐dependent protein lysine deacetylases with or without mono‐ADP‐ribosylase activity.^1^, ^2^ They are expressed in both small organisms (e.g., yeast and bacteria) and large mammals (e.g., humans) and share an evolutionarily conserved catalytic region of 275 residues while differing in the size and sequence of their N‐ and C‐terminal domains, which define their substrate specificity, the reaction they catalyzed, and subcellular localization.^3^ SIRTs play crucial physiological roles in various tissues and organs including the skin, muscles, skeleton, adipose tissue, lymphatic system, blood, nervous system, lungs, liver, kidneys, pancreas, heart, prostate, and ovaries, among others.^4^ Each sirtuin homolog binds to specific molecular targets, generating unique biological effects in the organs in which they are expressed.

Several issues of the Journal of Cell Communication and Signaling (JCCS) have featured studies highlighting the critical role of SIRT1 in cardiovascular disease models. Yin et al. identified a mechanistic link between SIRT1, the gut microbiota‐derived metabolite trimethylamine N‐oxide/TMAO, and the inflammatory response in vascular smooth muscle cells.^5^ Their findings suggest that SIRT1 may attenuate atherosclerotic plaque formation and slow disease progression by dampening inflammation and modulating SM22α expression. Begum et al.’s study suggested that the anti‐inflammatory, antioxidant, and wound healing properties of dehydrozingerone can be attributed, at least in part to the upregulation of SIRT1.^6^ In a separate study, Liu et al. identified a novel diabetic retinopathy (DR)‐associated circular RNA, cPWWP2A that functions as a competing endogenous RNA by sequestering miR‐579.^7^, ^8^ Through this interaction, cPWWP2A promotes DR‐associated retinal vascular dysfunction by upregulating the expression of multiple target genes, including SIRT1. These studies exemplify a growing body of research exploring the regulation, biological functions, and therapeutic potential of SIRT1‐based interventions. Given this expanding evidence, several critical questions arise: What are the precise molecular mechanisms by which SIRT1 exerts its effects? Can SIRT1 be reliably targeted in pathological conditions as a therapeutic strategy? How does the timing and context of SIRT1 activation or inhibition influence disease initiation and progression? Finally, to what extent can preclinical findings on SIRT1 be translated into clinical applications?

SIRT1 is the most studied sirtuin mainly for its ubiquitous expression and its involvement in the regulation of a wide range of cellular responses.^9^ In mammalian cells, SIRT1 promotes deacetylation of histone H1 at lysine 26, histone H4 at lysine 16, and H3 at lysine 9 and K79.^10^ The latter also promotes DNA hypomethylation. Deacetylation of histone tails and spreading of hypomethylated H3‐K79 favors the formation of tightly compacted chromatin and gene silencing, which is a hallmark of SIRT1 molecular activities. Because histone deacetylation is associated with decreased chromatin accessibility, histone deacetylase activity for SIRT1 was directly associated with the regulation of telomeric chromatin,^11^ gene expression, and the dynamic chromatin association of DNA repair factors.^12^

Telomere shortening is a deleterious processes that occurs over the lifespan and has been linked to the concomitant decrease of SIRT1 expression with age.^13^, ^14^ Similarly, age‐related DNA hypomethylation linked to reduced DNA methyltransferase activity have been correlated with reduced SIRT1 activity and decreased longevity.^15^ However, the interplay between SIRT1 expression and the rate of aging remains outstanding. The best studies highlighting a cause and effect relationship between SIRT1 expression and longevity stemmed from observations made in lower organisms as genetic overexpression of SIRT1 was linked to increase lifespan of yeast,^16^ worms,^17^ and flies.^18^ In mice, earlier studies by Kenyon et al. and Bonkowski et al. showed that overexpression of the Sirt1 gene led to beneficial phenotypes that may be relevant to human health and longevity.^19^, ^20^ Studies by several groups showed that mice engineered to overexpress SIRT1 or express additional copies of SIRT1 have improved organ function, increased disease resistance, and longevity.^19^, ^21^, ^22^, ^23^ A transgenic mouse with high levels of SIRT1 exhibited beneficial metabolic effects including reduced blood lipid levels and improved glucose metabolism while a transgenic mouse with moderate overexpression of SIRT1 was resistant to inflammation, and was protected from liver cancer, diabetes, and hepatic steatosis.^24^, ^25^ Yet, the latter SIRT1 transgenic mouse strains did not have a longer lifespan. These apparent discrepancies among different studies can be explained by the potential contribution of environmental or dietary factors such as calorie restriction regimen and perhaps, general approach towards SIRT1 activity regulation (ubiquitous expression versus tissue‐specific and tissue‐selective regulation). Thus, the initial presumption that SIRT1 activity correlates with extension of longevity, whereas it pertains to organisms such as worms and flies, it may not necessarily apply to mammals which somewhat argues against simplistically and systematically extrapolating research findings from lower organisms to mammals. One cannot rule out the idea that SIRT1 activity alone may not be sufficient to influence lifespan and that other intrinsic or extrinsic environmental factors may synergize or antagonize SIRT1 activity overtime in mammals.

Although the longevity benefits of SIRT1 overexpression remain controversial, SIRT1 deficiency has been consistently linked to an increased risk of age‐related diseases and heightened susceptibility to metabolic disorders.^26^ Aging is accompanied by a progressive decline in SIRT1 activity, leading to reduced expression of core circadian clock components and disruption of central circadian regulation.^27^, ^28^ This disruption is considered an early hallmark of premature aging and is implicated in the pathogenesis of metabolic and neurodegenerative diseases. In the liver, SIRT1 deacetylates PGC‐1α and amplifies the expression of the circadian transcription factors BMAL and CLOCK in the suprachiasmatic nucleus of the hypothalamus.^29^, ^30^ A key transcriptional target of the core circadian regulators BMAL1 and CLOCK is NAMPT, the rate‐limiting enzyme in the NAD^+^ salvage pathway from nicotinamide.^31^ As a result, NAD^+^ is synthesized in a circadian oscillatory manner throughout the body, establishing a rhythmic pattern of SIRT1 activation and deactivation in alignment with the circadian cycle.

The study published in JCCS by Yin et al. on the role of SIRT1 in influencing the inflammatory effects of gut microbiome and the ensuing development of atherosclerotic lesions in preclinical model of atherosclerosis is corroborated by other studies emphasizing the SIRT1 effects on metabolism and metabolic syndrome.^5^, ^32^ SIRT1 transgenic mice were protected from diabetes and diet‐ or genetically induced obesity.^25^, ^33^ Similarly, transgenic mice with constitutive expression of SIRT1 are resistant to developing liver steatosis whereas their heterozygous counterparts are prone to developing this disease.^34^ Paradoxically, another study showed that SIRT1‐null mice presented higher glucose tolerance and protection from fatty liver induced by LXR agonists.^35^ It should be noted that polymorphisms of SIRT1 play a role in the susceptibility to type 2 diabetes mellitus and the development of vascular complications of diabetes.^36^ Zhang et al. demonstrated that endothelium‐specific overexpression of SIRT1 decreases atherosclerosis in apolipoprotein E‐deficient mice.^37^ Other studies provided additional evidence that activation of SIRT1 can improve metabolic disturbances of cardiomyocytes and effectively inhibit their massive loss in the heart.^38^

Mechanistically, the diverse biological roles of SIRT1 in the cardiovascular system are attributed to its interaction with multiple molecular targets. SIRT1 functions as a deacetylase, modulating over 40 known substrates involved in a variety of pathways, including inflammation, stress resistance, mitochondrial biogenesis, fatty acid metabolism, insulin secretion, glucose production, and lipid homeostasis.^39^, ^40^ Its targets include, among many others, PPAR‐γ and its coactivator PGC‐1α, protein tyrosine phosphatases, FOXO transcription factors, AMP‐activated protein kinase, CREB‐regulated transcription coactivator 2, endothelial nitric oxide synthase, p53, myogenic differentiation factor, liver X receptor, and transcription factor E2F1.^41^, ^42^

SIRT1 exerts antioxidative effects, notably through the deacetylation of p53, which upregulates manganese superoxide dismutase expression.^43^ In cardiomyocytes, SIRT1‐mediated p53 deacetylation downregulates pro‐apoptotic proteins Bax and caspase‐3, thereby reducing apoptosis, an effect especially relevant in the border zone following myocardial infarction.^44^, ^45^ Additionally, SIRT1 modulates FOXO transcription factors, shifting their activity from promoting apoptosis to enhancing resistance to oxidative stress and promoting autophagy, thus supporting cell survival.^46^, ^47^ A positive feedback loop has been identified between SIRT1 and FOXO1: SIRT1 deacetylates FOXO1, enhancing its transcriptional activity, whereas FOXO1, in turn, activates SIRT1 expression by binding to a regulatory site in the SIRT1 promoter. These findings underscore the tissue‐ and organ‐specific effects of SIRT1, which may not always be apparent at the whole‐organism level.

Despite controversial findings about SIRT1 effects in different experimental animal models, the molecular effects of SIRT1 as a promoter of DNA damage repair, cell survival, and metabolism, and control of oxidative stress and inflammation support a rather protective role of SIRT1 against metabolic diseases.^48^ A race to identify SIRT1 modulators ensued with the prospect to treat various diseases and maintain physiological balance.^49^ These efforts lead to the identification of several compound polyphenols as SirTuin‐activating compounds (STACs) including resveratrol (RSV), flavonoids, terpenes, ellagic acid, quercetin, anthocyanins, and curcumin.^50^ RSV aka 3,5,4′‐trihydroxystilbene, which is commonly found in a wide variety of fruits, vegetables and nuts stood out as particularly representative of the STACs.^51^ RSV is a highly lipophilic and poorly water soluble molecule.^52^ It has a high intestinal absorption and undergo a rapid metabolization by the gut microbiota.^53^ The plasma concentration of RSV metabolites increases up to 20‐fold immediately after the administration of high doses of RSV. After absorption, RSV accumulates in the liver where it was found bound to serum albumin and low‐density lipoproteins.^54^ Therefore, biological effects of RSV are surmised to be linked to the activities of its metabolites, reconversion of metabolites to RSV in organs and tissues, recirculation pathways, or all of the aforementioned processes. Molecularly, RSV inhibits phosphodiesterase which increase cAMP levels, and intracellular calcium and subsequent activation of the AMPK pathway and elevation of NAD^+^, a cofactor required for SIRT1 activation.^55^ As a result, RSV was able to recapitulate several SIRT1 activities by modulating cellular signaling pathways such as NF‐κB, p53, AMPK, mTOR, and JAK2/STAT3, leading to reduced expression of pro‐inflammatory and pro‐angiogenic factors such as TNF‐α, IL‐6, COX, HIF‐1α, and VEGF.^40^, ^56^ At the same time, it enhances PARP1 activity, upregulates antioxidant enzymes including catalase, superoxide dismutase, and glutathione peroxidase.^57^, ^58^, ^59^ Many of these pathways overlap with SIRT1 signaling, making it challenging to distinguish between sirtuin‐dependent and independent actions of RSV.^60^ In preclinical models of cardiovascular diseases, RSV administration provided anti‐inflammatory, antioxidant, hepatoprotective, cardioprotective, anti‐obesity, and anti‐diabetic effects.^61^ In the ischemia/reperfusion rat model, RSV modulated inflammation and oxidative damage by inducing Nrf2/ARE activation through blockage of Keap1.^62^, ^63^ RSV also prevents myocardial infarction by reducing myeloperoxidase and MDA levels in the myocardium as well as serum lactate creatinine kinase and dehydrogenase levels and raising superoxide dismutase and GPx activity.^64^

Similarly, RSV shows protective effects against vascular occlusive disease by inhibiting neointima formation and enhancing endothelial function.^65^, ^66^ It does so primarily through the activation of the Nrf2/HO‐1 pathway, which boosts antioxidant defenses and reduces oxidative stress. RSV helps counteract mitochondrial dysfunction and oxidative damage caused by high glucose levels.^67^, ^68^ In mice fed with a high‐fat diet, RSV restores Nrf2‐dependent vasodilation, reduces oxidative stress and apoptosis in femoral arteries, and improves endothelial function.^69^, ^70^ Additionally, in a mouse model of atherosclerosis, RSV inhibits the expression of ICAM‐1, a key factor involved in monocyte adhesion, through the modulation of Nrf2/ARE signaling.

It should be noted that by regulating cellular processes such as DNA repair, cell cycle, autophagy, mitophagy, metabolic regulation, inflammation, and oxidative stress control, SIRTs also regulates tumorigenesis.^71^, ^72^ These effects are highly context‐dependent and its role in tumor suppression and growth remains controversial.^73^, ^74^ SIRT1 was found to be up‐regulated in breast, prostate, skin, lung, colon, and liver cancer as well as in lymphomas and leukemia and was suggested to promote cancer cell growth, survival, and invasiveness^75^, ^76^. Likewise, chemotherapy and drug resistance have been linked to SIRT1 upregulation.^77^, ^78^ SIRT1 induces deacetylation of tumor suppressor genes like p53.^78^, ^79^ As a result, p53 loses its tumor suppressor function and increases cancer cell growth while inhibiting their apoptosis. Therefore, inhibiting SIRT1 protein activity may prevent loss of p53 function and ultimately exert antitumorigenic effect. Because the use of SIRT1 inhibitors could be an effective epigenetically regulated anticancer treatment approach, a series of synthetic compounds that inhibit SIRT1 activity have been developed.^80^ Sirtinol was the first cell‐permeable SIRT1 inhibitor identified in 2003 that effectively inhibits NAD^+^‐dependent deacetylase activity of SIRT1.^81^, ^82^ Splitomicin, another SIRT1 inhibitor with potential application in cancer therapy has been later identified.^83^ The efficiency of other SIRT1 inhibitors such as tenovin‐1, tenovin‐2 and salermide among others require further experimental and preclinical validation.^84^

However, many aspects of SIRT1 in tumor growth are still ambiguous and controversial. Conflicting evidence suggests SIRT1 can also act as a tumor suppressor by inhibiting beta‐catenin and NF‐kappaB signaling.^85^, ^86^ Along these lines, RSV has demonstrated anticancer effects, as evidenced by its ability to inhibit the growth of a broad range of tumor cells.^87^ These include cancers of the lymphoid and myeloid lineages, multiple myeloma, and malignancies of the breast, prostate, stomach, colon, pancreas, and thyroid, as well as melanoma, head and neck squamous cell carcinoma, ovarian carcinoma, and cervical carcinoma.^88^, ^89^ Its growth‐suppressing activity is associated with its propensity to induce cell‐cycle arrest, increase the expression of p21/Cip1/WAF1, p53, and Bax, and decrease the levels of survivin, cyclin D1, cyclin E, Bcl‐2, Bcl‐xL, cIAPs, and caspases.^90^, ^91^ Overall, a better understanding of the biological chemistry of SIRT1 activators and inhibitors as well as their off target effects will pave the road for their potential therapeutic usefulness in therapeutic contexts.

## 2 CONCLUSIONS AND FUTURE PERSPECTIVES

SIRT1 plays pivotal regulatory roles in cellular processes such as apoptosis inhibition, aging delay, oxidative stress response, inflammation control, and tumor progression. Increasing evidence supports the therapeutic relevance of SIRTs modulators in a range of diseases, including cancer, cardiovascular, autoimmune, and neuroendocrine disorders. Although several well‐characterized modulators such as RSV and SRT2104 have demonstrated promise, many novel agents remain at the preclinical stage due to challenges related to specificity, bioavailability, safety, and clinical translation. Future research should prioritize the design of more selective and potent modulators, improve delivery systems, and explore combination therapies to enhance therapeutic efficacy. Additionally, integrating genomics and biomarker profiling into clinical strategies could facilitate the development of personalized treatment regimens. Interdisciplinary collaboration among researchers in biology, pharmacology, genetics, and clinical medicine will be essential to overcome current limitations and fully realize the clinical potential of SIRT1 modulators.

Importantly, the role of SIRT1 in neurovascular and neurodegenerative diseases remains incompletely understood and warrants further comprehensive investigations, particularly given its involvement in neuronal survival, vascular integrity, and metabolic regulation.^92^, ^93^ A deeper mechanistic understanding of SIRT1 function within the neurovascular unit will be critical for identifying disease‐relevant targets and therapeutic windows. In parallel, the development of tailored context‐dependent strategies to pharmacologically modulate SIRT1 activity in these systems will be essential to maximize therapeutic benefit while minimizing off‐target effects.

Integration of biomarker profiling, multi‐omics, and system‐level approaches into clinical research may further enable personalized therapeutic strategies. Interdisciplinary collaboration across biology, pharmacology, genetics, and clinical research will be key to overcoming current challenges and advancing SIRT1‐targeted interventions. As precision medicine evolves, refined modulation of SIRT1 and related pathways holds considerable promise for improving disease prevention and treatment outcomes.

## CONFLICT OF INTEREST STATEMENT

Dr Chaqour is the Editor‐in‐Chief of JCCS.

## ETHICS STATEMENT

Not applicable.

## CONSENT FOR PUBLICATION

Not applicable.

## Data Availability

Data sharing not applicable to this article as no datasets were generated or analyzed during the current study.
